# Computation of geographic variables for air pollution prediction models in South Korea

**DOI:** 10.5620/eht.e2015010

**Published:** 2015-10-23

**Authors:** Youngseob Eum, Insang Song, Hwan-Cheol Kim, Jong-Han Leem, Sun-Young Kim

**Affiliations:** 1 Department of Geography, Seoul National University, Seoul, Korea; 2 Department of Geography, State University of New York at Buffalo, NY, USA; 3 Department of Occupational and Environmental Medicine, Inha University School of Medicine, Incheon, Korea; 4 Institute of Health and Environment, Seoul National University, Seoul, Korea

**Keywords:** Air pollution, Cohort study, Exposure prediction, Geographical information system

## Abstract

Recent cohort studies have relied on exposure prediction models to estimate individuallevel air pollution concentrations because individual air pollution measurements are not available for cohort locations. For such prediction models, geographic variables related to pollution sources are important inputs. We demonstrated the computation process of geographic variables mostly recorded in 2010 at regulatory air pollution monitoring sites in South Korea. On the basis of previous studies, we finalized a list of 313 geographic variables related to air pollution sources in eight categories including traffic, demographic characteristics, land use, transportation facilities, physical geography, emissions, vegetation, and altitude. We then obtained data from different sources such as the Statistics Geographic Information Service and Korean Transport Database. After integrating all available data to a single database by matching coordinate systems and converting non-spatial data to spatial data, we computed geographic variables at 294 regulatory monitoring sites in South Korea. The data integration and variable computation were performed by using ArcGIS version 10.2 (ESRI Inc., Redlands, CA, USA). For traffic, we computed the distances to the nearest roads and the sums of road lengths within different sizes of circular buffers. In addition, we calculated the numbers of residents, households, housing buildings, companies, and employees within the buffers. The percentages of areas for different types of land use compared to total areas were calculated within the buffers. For transportation facilities and physical geography, we computed the distances to the closest public transportation depots and the boundary lines. The vegetation index and altitude were estimated at a given location by using satellite data. The summary statistics of geographic variables in Seoul across monitoring sites showed different patterns between urban background and urban roadside sites. This study provided practical knowledge on the computation process of geographic variables in South Korea, which will improve air pollution prediction models and contribute to subsequent health analyses.

## Introduction

Cohort studies have investigated associations between longterm exposures to air pollution and various health outcomes based on the spatial contrasts of exposures and outcomes of individuals [1,2]. A significant challenge in these studies is that individual measurements of air pollution are not available. Some recent studies have developed exposure prediction approaches to estimate individual-level concentrations of air pollution [3,4]. Geographic variables, computed by using geographic information system (GIS), were often included in such models as predictors for air pollution concentrations at given locations. These variables represented potential sources of air pollution such as traffic and population in surrounding areas and improved the characterization of fine-scale variability of air pollution [3]. Large cohort studies in North America and Europe computed hundreds of variables to represent possible sources [5,6].

Interest has increased in estimating air pollution exposures based on cohort studies in South Korea [7,8]. However, most exposure prediction approaches have relied only on air pollution monitoring data without incorporating geographic variables, possibly due to logistical constraints [9]. The computation of geographic variables requires extended knowledge of available data sources and GIS techniques.

In the present study, we aimed to develop a list of geographic variables based on their relationships with air pollution reported in previous studies. Subsequently, we intended to explore available data sources, to combine all data to a single GIS database and to compute the finalized variables at 294 regulatory air pollution monitoring sites in South Korea. Specifically, we considered various geographic variables as input data for statistical exposure prediction models that have been largely used in cohort studies of air pollution. Our work focused on 2010 data owing to the large number of monitoring sites and availability of the most recent census data [10]. The data integration and variable computation were performed by using ArcGIS version 10.2 (ESRI Inc., Redlands, CA, USA).

## Conceptual Background of Geographic Variables

We explored eight categories of geographic variables as potential sources of air pollution. The choice of eight categories was based largely on two large cohort studies, the European Study of Cohorts for Air Pollution Effects [5] and the Multi-ethnic Study of Atherosclerosis and Air Pollution [6], which examined particulate matter (PM) less than or equal to 10 μm and 2.5 μm in diameter (PM_10_ and PM_2.5_, respectively) in addition to black carbon, nitrogen oxide (NO_X_), nitric oxide (NO), and nitrogen dioxide (NO_2_) air pollution. The eight categories include traffic, demographic characteristics, land use, transportation facilities, physical geography, emissions, vegetation, and altitude (Table 1). Each variable was calculated at regulatory monitoring sites by using one of two metrics: the distance to a feature or a buffer summary statistic (e.g., sum or proportion) of a feature. A buffer is a circular feature that indicates that the air pollution concentration measured at the central point of the buffer is influenced by probable sources at a given distance. In this study, we used three sets of different buffer sizes. The buffer radii for traffic variables were 25, 50, 100, 300, 500, and 1000 m, whereas larger radii of 100, 300, 500, 1000, and 5000 m were applied to nontraffic variables for demographic characteristics and land use categories [5,11,12]. In addition, the largest buffer radii of 3, 15, and 30 km were adopted for emission variables [13].

### Traffic

Because traffic is considered a major source of air pollutants such as PM_2.5_ and NO_X_, associated with health endpoints in epidemiological studies, geographic variables reflecting traffic have been widely used in exposure prediction models in many cohort studies [14-17]. Vehicles emit various air pollutants from exhaust and non-exhaust factors such as diesel/gasoline engines, brake wear, and road surface wear. The amount of traffic estimated on each road for a given time period could be the best metric for representing vehicle emission. However, traffic volume data are not generally available [12]. Instead, the proximity to the nearest roads and lengths of surrounding roads are frequently adopted as proxies for traffic volume. Road lengths are summed within specific buffer sizes. In particular, NO_X_, NO, and NO_2_ have been positively associated with decreasing distances to major roads and increasing lengths of roads in surrounding areas [3].

### Demographic Characteristics

Densities of population and households implying human activities related to heating, cooking, and transportation would result in increasing air pollution concentrations. The increasing number or density of residents and households in given buffer areas tended to be related to the elevated levels of air pollution concentrations in Europe, Canada, the US, and Taiwan [5,12,18-20].

### Land Use

The types of land use such as residential, urban, and green areas have been used as significant predictors for PM and NO_X_/NO_2_ air pollution in North America and Europe [3]. A study in New York City used a vegetative land use variable to explain the variation in PM_2.5_ concentrations [17]. In addition, a study in Taiwan using five land use categories found that high proportions of urban green and natural areas in given buffers were associated with decreased NO_X_ concentrations, whereas a high proportion of the low density residential area contributed to increased NO_2_ [12].

### Transportation Facilities

Public transportation depots such as airports, ports, subway stations, and bus stops could affect increased air pollution concentrations owing to the high emission from transportation equipment and facilities. For example, a large number of concentrations of ultrafine PM and PM_2.5_ was found to be related to aircraft takeoff [21]. PM_2.5_ concentrations attributable to aviation emissions were shown to decrease with increasing distances from airports in the UK [22]. Moreover, a study in Italy reported high concentrations of NO_2_ near a port area, possibly attributed to vessel traffic emission [23].

### Physical Geography

Natural geographical features may also affect air pollution. For example, the proximity to water bodies affecting air flow in river valleys and oceans could be related to air pollution. The remoteness to a coastline has been associated with increasing NO_2_ in San Diego, California, the US [24] and decreasing PM_8_ in Shizuoka Prefecture, Japan [25].

### Emissions

Emissions derived from various sources for major pollutants could be related to air pollution concentrations. Many countries have provided emission estimates of major pollutants from pollution sources such as roads, transportation, industry, and agriculture [26, 27]. To improve exposure prediction models, some studies in the US and Italy have included emission estimates for primary pollutants [6,28].

### Vegetation

Normalized Difference Vegetation Index (NDVI), one of the most frequently used vegetation indices, measures the density of green vegetation on land by using satellite images. This index is computed by determining the reflectance values on a target area of the earth’s surface in the visible red (RED) and near-infrared (NIR) bands, as shown in equation 1 [29].

(1) $$\frac{\left(NIR-RED\right)}{\left(NIR+RED\right)}$$

High NDVI values imply abundant vegetation and may be negatively associated with air pollution levels. Studies of exposure prediction models in the US have used medians and quantiles of 16-day composite NDVI values over one year and seasonal values for high-vegetation and low-vegetation seasons [6].

### Altitude

Atmospheric pressure and circulation changes by elevation could affect movements of air pollutants and air pollution concentrations at a given location. Although altitude has been negatively associated with PM_2.5_ concentrations in four European cities of substantially varied altitudes [5], one study in Taipei, Taiwan, with relatively homogenous altitudes across monitoring sites excluded altitude variables in the final exposure prediction model for NO_X_ and NO_2_ [12].

## Data Acquisition

### Locations of Regulatory Monitoring Sites

The Ministry of Environment in South Korea established regulatory air quality monitoring networks to monitor the conditions of air pollution and attain the air quality standards since 1980s [30, 31]. We obtained the addresses and coordinates of the 294 monitoring sites operated nationwide in 2010 from the Annual Report of Ambient Air Quality in Korea 2010 [31]. When address and coordinates did not match, we used the address and extracted coordinates from Google Maps.

### Road Networks

Road network data for 2010 were obtained from the Korean Transport Database (KTDB) of the Korea Transport Institute (http://www.ktdb.go.kr). The shapefile, a popular data file format in GIS software, for road networks is composed of more than 100000 line segments and corresponding characteristics of each road segment such as a road name, speed limit, direction, and number of lanes. KTDB classifies all roads in South Korea into eight types including national highway, metropolitan city highway, general national road, metropolitan city road, government- financed provincial road, provincial road, district road, and highway link lamp. In addition, the monthly data for the number of registered vehicles in 2011 for a district administrative unit with a median area of 391 km_2_ in 2010, known locally as a si–gun–gu, were downloaded from the Korean Statistical Information Service website of the Statistics Korea (http://kosis.kr/eng/); 2010 is the earliest year with available data.

### Census

We obtained 2010 census tabular data for a census territorial unit, as a census output area, known as a jipgegu with a median area of 0.02 km_2_ in 2010, from the Statistical Geographic Information Service (SGIS) of the Statistics Korea (http://sgis.kostat.go.kr). For each jipgegu, the numbers of residents, households, housing buildings, companies, and employees were available as a total value and by classification of gender and age, type and construction year of houses, and type of business. The type of house was classified on the basis of the size and height of the housing building, whereas the type of business was based on the seven categories of the Korean standard industrial classification. We also obtained a shapefile of jipgegus from the SGIS.

### Land Cover Map

Land cover maps were obtained from the Environmental Geographic Information Service of the Korea Ministry of Environment (http://egis.me.go.kr). Land surface images captured by satellites were converted into land cover maps consisting of areas with various land surface characteristics categorized by using image-adjustment and image-classification algorithms. To cover the entire country through 2007, 814 maps were created; 150 maps mostly the Seoul metropolitan areas were updated in 2009. To use the most recently updated maps, we selected the 2007 maps and replaced 150 areas with the updated 2009 data. These land cover maps consisted of 7, 22, and 41 classes for high, medium, and low spatial levels, respectively. The seven high-level classes included urbanized and built areas, agricultural areas, forest areas, grasslands, wetlands, bare ground, and waters (Table 2). We used the high-level classes except for the urbanized and built area class. For such class, we replaced by seven medium-level classes reflecting specific land use characteristics possibly associated with air pollution in complex urban settings.

### Emissions

We downloaded the tabular data for emissions, created by the National Institute of Environmental Research, from the National Air Pollutants Emission website (http://airemiss.nier.go.kr/main.jsp). The emission data contain annual emission estimates of seven pollutants including carbon monoxide, NO_X_, sulfur oxide, total suspended particulates, PM_10_, volatile organic compounds, and ammonia aggregated on a 1-km grid by point, line, and area sources with grid coordinates indicating the bottom left corner of each grid cell.

### Normalized Difference Vegetation Index

For NDVI, we downloaded satellite images from the Institute of Industrial Science (IIS), University of Tokyo (http://webmodis.iis.u-tokyo.ac.jp/). The IIS provides cloud- and shadowfree images captured by Aqua/Terra Moderate Resolution Imaging Spectroradiometer (MODIS) satellites over Asia every 10 days. The 10-day composite images were created by using the Enhanced Second Minimum composition method, which selects a pixel with second minimum reflectance in the red channel as a representative value during a 10-day period to avoid pixels shadowed by clouds [32]. The spatial resolution of these raster image data was approximately 428 m for each cell. The NDVI pixel image values were between 0 and 255.

### Other data

Shapefile data for bus stops were downloaded from the Biz-GIS website (http://www.biz-gis.com/XsDB/). This website, operated by the Biz-GIS company, provides spatial data in South Korea in shapefile formats for various spatial features such as apartments, hospitals, banks, and retail establishments. The addresses and coordinates of airports generated by the Korea Airports Corporation were obtained from an open data portal (https://www.data.go.kr) that offers data generated by various public agencies. Coastline data generated by the Korea Hydrographic and Oceanographic Administration were obtained from the National Spatial Information Clearinghouse (https://www.nsic.go.kr/ndsi/). We obtained shapefile data for railroad stations, subway stations, urbanized areas, rivers, and boundaries of si–gun–gu from the SGIS. The Shuttle Radar Topography Mission (SRTM) 30 m × 30 m digital elevation model (DEM) data for South Korea was downloaded from the EarthExplorer interface of the United States Geological Survey website (http://earthexplorer.usgs.gov).

## Data Integration

To compute the geographic variables, we integrated all data obtained from the various sources into the one GIS database. This integration included the transformation of different coordinate systems to a single system. Spatial data generated from the various organizations were on different coordinate systems or no information was provided for coordinate systems. This limitation created difficulties in displaying all available data on the same map, which is necessary for computing geographic variables. Thus, we adopted the Korean Central Belt 1995 coordinate system, which is most commonly used in our GIS data (Table S1).

In addition, we combined tabular, vector, and raster data into the database. The data for monitoring sites, census, registered vehicles, and emissions were provided in tabular formats. Shapefiles for road networks and administrative boundaries included vector data representing point, line, and polygon features. Image files for satellite data contained raster data, which display data values on grid cells.

## Variable Computation

### Traffic

We computed the distance to the nearest road, the sum of road lengths in a buffer space, and the number of registered vehicles at each regulatory monitoring site. The distance to the nearest road was computed as the minimum Euclidian distance between the line of the road and each monitoring site. For the sum of road lengths, we created various sizes of traffic buffers, selected road segments within each buffer, and aggregated the lengths of all selected road segments. For the number of registered vehicles, we calculated the annual average of registered cars from monthly data for each si–gun–gu. Then, we linked this tabular data to the shapefile of si–gun–gu and identified the number of vehicle registrations in the si–gun–gu that included a target monitoring site.

**Figure 1.**
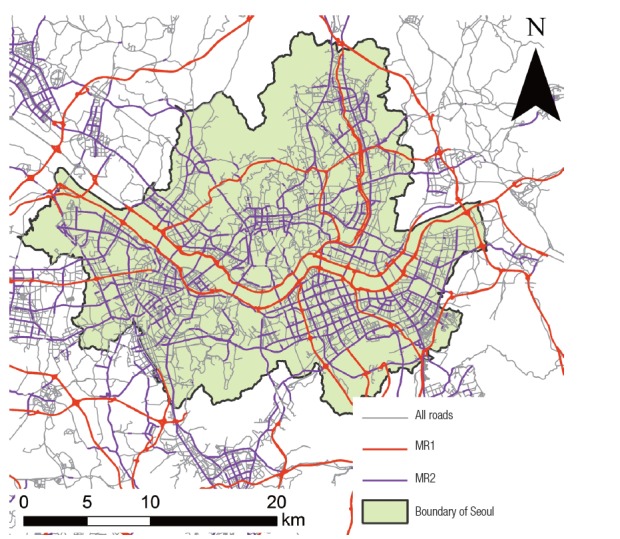
Road networks in Seoul, Korea. MR1, major road 1; MR2, major road 2.

The traffic variables of road networks were computed for each of the three categories of roads including all roads, major road 1 (MR1), and major road 2 (MR2). MR1 was defined by national highways and metropolitan city highways, whereas MR2 included MR1 as well as local roads with more than six lanes (Figure 1). We created the category of MR2 owing to the limited number of MR1 roads. The total length of all roads was 90816 km in South Korea, whereas lengths of MR1 and MR2 were 8128 and 12194 km which are 8.95 and 13.43 % of all roads, respectively.

In addition, we incorporated the numbers of lanes and road widths to the sum of road length variables. Road networks in the KTDB are represented by single line features of the centerlines of roads without considering lanes and widths. The KTDB road network data contains attributes of the number of lanes. We assigned road width values depending on highway/nonhighway, speed limit, and urban/non-urban areas based on the Administrative Rule on the Structure and Installation of Road (Table S2) [33]. Information on highway/non-highway and speed limit were given in the KTDB road network data. For urban/ non-urban areas, we defined the urban area that overlapped with the urbanized area shapefile obtained from the SGIS (Figure S1). Thus, the road segments intersecting with urbanized areas were considered as roads in the urban areas; others were defined as non-urban roads.

### Demographic Characteristics

**Figure 2.**
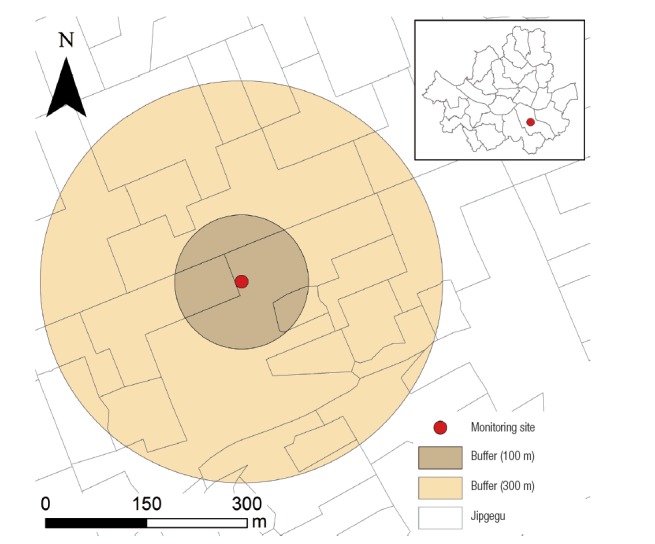
Map of 100 and 300 m buffers and nearby jipgegus of a regulatory monitoring site in Seoul, Korea.

We computed the numbers of total population and households, numbers of housing buildings by construction years and house types, and numbers of companies and employees by business types in a non-traffic buffer. After linking the tabular census data to the administrative boundary shapefiles by using the jipgegu identifier, we selected jipgegus intersecting with a nontraffic buffer and aggregated the numbers of residents, households, housing buildings, companies, or employees within intersected jipgegus with the weight of jipgegu sizes (Figure 2). For example, equation 2 shows the total population within a given buffer i (P*_i_*), derived by using total population in a jipgegu j (P*_j_*) and an areal weight as the ratio of the area of the intersected jipgegu with the buffer (A*_ij_*) to the area of the jipgegu (A*_j_*).

(2) $$P_{i}=\sum_{j=1}^{N}\frac{P_{j}\;\times \;A_{ij}}{A_{j}}$$

### Land Use

A land use variable was computed as the proportion of the areas for a type of a land use to the area of a given non-traffic buffer. For each type of the 12 land use classes within a buffer area, we selected the polygons of each land use within the buffer and computed the proportions of the selected areas of the land use to the buffer area.

### Transportation Facilities

The distances of a monitoring site to the nearest transportation depots such as railroad stations, subway stations, bus stops, airports, and major ports were calculated. We defined major ports by ports that accommodate more than 10000 vessels per year based on the statistics from the Shipping and Port Integrated Data Center. Ten out of 31 ports were identified as major ports in South Korea.

### Physical Geography

The minimum distances to rivers, coastlines, and the border between North and South Korea were computed. To produce the borderline, we combined all jipgegu polygons into a single polygon, converted the polygon into a line feature representing the outer boundary of South Korea, and extracted the northern end.

### Emissions

By using the emission tabular data at 106070 1-km national grid coordinates in South Korea, we aggregated emission estimates from point, line, and area sources at each coordinate. Then, we created 1-km grid-shaped polygons based on grid coordinates, and assigned emission estimates at the grid points to corresponding polygons. Air pollutant emissions in grid polygons were accumulated within 3, 15, and 30 km buffers with areal weight as previously described in the demographic characteristics.

### Normalized Difference Vegetation Index

We computed the average, minimum, and maximum of 36 10 day composite MODIS NDVI data during 2010 for each grid to avoid seasonal variation and to estimate spatially representative values. In addition, the median for August during 2009, 2010, and 2011 was computed for reflecting the lushest vegetation in South Korea. Finally, we extracted the NDVI values at each monitoring site from the grid in which the monitoring site is located.

### Altitude

By using the 30 m×30 m SRTM DEM raster image data, we assigned the elevation value in a grid cell to each monitoring site included in the cell. In addition, we computed the relative elevation as the proportion of concentric cells in which the elevation values are above or below threshold elevations of 20 and 50 m, respectively, compared with that at a monitoring site. The concentric cells refer to the DEM grid cells on a 30 m-wide donutshaped polygon 1 or 5 km from the monitoring site.

## Summary of Computed Variables

On the basis of the 313 geographic variables computed at 294 regulatory monitoring sites in South Korea, we examined the relationships between traffic variables and the summary statistics of selected variables to verify our computation results and to provide insight into distributions of the geographic variables. Our presentation of descriptive statistics was restricted to Seoul and focused on the comparison by two types of regulatory monitoring sites including 25 urban background and 12 urban roadside sites.

**Figure 3.**
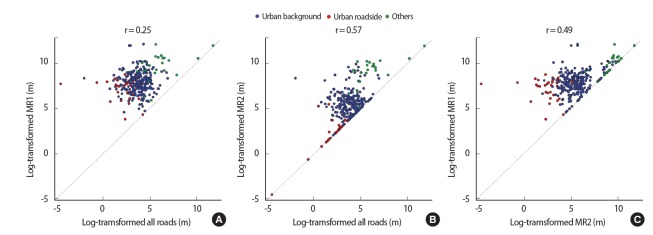
Scatter plots between all roads and major road 1 (MR1) (A), between all roads and major road 2 (MR2) (B), and between MR1 and MR2 (C) across 294 monitoring sites in South Korea.

Figure 3 shows the relationships of natural log-transformed distances to the nearest road among all roads, MR1, and MR2 across regulatory monitoring sites in South Korea. For most monitoring sites, the nearest road was a local road rather than an MR1 or MR2. For one urban roadside site, a MR1 was the nearest road. This site is the only regulatory monitoring site located on a metropolitan city highway according to its address, indicating that our computation was accurate. For about 20 % of the sites, MR2 roads were the nearest roads.

Table 3 gives summary statistics of the two types of sites in Seoul. The urban roadside sites were located closer to all roads or MR2s than urban background sites and were more surrounded by these roads. The differences of the sums of road lengths within 300 m buffers between the two sites types increased when lanes and widths were applied. More than a half of the sites did not have an MR1 within 300 m. More residents lived within 300 m from urban ambient sites than urban roadside sites, and fewer workers were employed in construction, lodging, and restaurant businesses. The average proportion of residential areas within 300 m from urban roadside sites (25 %) was lower than that within 300 m from urban background sites (39 %). Emission estimates for PM_10_ within 3 km were consistent between the two types of sites (0.7 g/m^3^). The monitoring sites were located in relatively flat areas with less than 40 % of 5 km distant points below or above 20 m.

## Discussion

We demonstrated the computation process of 313 geographic variables at air pollution regulatory monitoring sites in the eight categories of possible pollution sources in South Korea. The computed variables reflected the geographic characteristics in South Korea and showed the different patterns between urban background and urban roadside sites.

For characterizing spatial variability of air pollution, we computed a large suite of geographic variables for the development of statistical exposure prediction models that rely largely on geographic variables. Previous studies included geographic variables chosen by model selection [34] or a few summary variables estimated by dimension reduction techniques [35] into statistical models. Whereas land use regression includes geographic variables only [3], universal kriging also incorporates the spatial correlation structure [36]. In addition to statistical methods, other studies have developed air quality models such as photochemical models and dispersion models based on the chemical and physical atmospheric processes of air pollution. These models used limited geographic variables of traffic, population, or emissions, and other input data such as meteorology. The different approaches resulted in inconsistent model performance in air pollution prediction [37-39] and varying health effect estimates in subsequent health analyses using predicted individual- level air pollution concentrations [40,41]. Studies comparing model performance between land use regression and dispersion models have generally showed large spatial variability in land use regression and large temporal variability in dispersion models [37-39]. Large spatial variability of air pollution is particularly important for assessing health effects of long-term exposures in cohort studies which largely rely on spatial contrasts. Because our ultimate goal in computing geographic variables lies in health analysis rather than the identification of air pollution distribution, we focused on statistical exposure prediction models and presented a large set of geographic variables.

Our descriptive statistics of geographic variables provided insights into data handling and model building in future studies of exposure prediction models. We found some extreme values particularly for distance variables. Some regulatory monitoring sites were located substantially far from national highways, airports, and ports. Variables with large variability resulting from extreme values could affect model selection and exposure prediction. Future studies should exclude or truncate such variables. We used small sizes of buffers including 25 m to represent the fine-scale spatial variability in a metropolitan city with high density. However, these small buffer variables may not provide meaningful or accurate values; few large roads were detected within 25 m and the distance to large roads based on central lines could contain errors similar to the buffer size. Future studies of exposure modeling approaches need to carefully consider the inclusion of these small-buffer variables.

We presented about 300 geographic variables that require future updates. Recent studies additionally included outputs of dispersion models and air quality models [42] or air pollution estimates determined by satellites [43] as predictors. Future studies need to introduce data sources and variable computation of these variables for South Korea. In addition, new data sources that have not been explored in previous studies mostly performed in North America and Europe may be available. These data could explain complex air pollution environments in other regions including densely populated metropolitan cities.

This study contributes to future studies of exposure prediction and health analyses. Our previous study in South Korea used ordinary kriging to predict air pollution concentrations based solely on spatial correlation without geographic variables. Findings showed poor model performance and suggested the inclusion of geographic variables to improve model performance [44]. An extended set of geographic variables could help explain the spatial variability of air pollution in complex urban environments in large and dense metropolitan cities in South Korea. A previous simulation study also showed that improved air pollution predictions tended to give less biased and more precise health effect estimates [45]. High quality exposure predictions incorporating geographic variables would clarify the association of air pollution and health in South Korea.

## Conclusion

The computation of extended geographic variables provides an opportunity for developing exposure prediction models that characterize heterogeneity of air pollution over space. This study will help future research utilize geographic variables for the development of prediction models and provide air pollution estimates with fine-scale spatial variability. Such air pollution predictions will allow subsequent health analyses based on individual exposure estimates in South Korea.

## Figures and Tables

**Table 1.** List of geographic variables in eight categories with their data sources and types of data

Category^a^	Variable	Source	Type of data (data format)
Traffic	Distance to the nearest roads (all roads, MR1, and MR2)	KTDB	Road network (line)
	Sum of road lengths (all roads, MR1, and MR2)^b^		
	Number of registered vehicles	KOSIS	Vehicle registration (table)
Demographic characteristics	Number of people	SGIS	Census (table)
	Number of households		
	Numbers of housing buildings by a type of residence and by a constructed year		
	Numbers of companies and employees by a type of business		
Land use	Proportions of residential, industrial, commercial, cultural, transportation, public facility, agricultural, forest, grassland, wetland, bare ground, and water areas	EGIS	Land cover map (polygon)
Transportation facilities	Distances to the nearest railroad and subway station	SGIS	Railroad and subway stations (point)
	Distance to the nearest bus stop	Biz-GIS	Bus stop (point)
	Distance to the nearest air port	ODP	Airport (table)
	Distance to the nearest major port	SP-IDC	Port (table)
Physical geography	Distance to river	SGIS	River (polygon)
	Distance to coastline	NSIC	Coastline (line)
	Distance to the military demarcation line	SGIS	Administrative boundary (polygon)
Emissions	Proportions of major pollutants (CO, NO_x_, SO_x_, TSP, PM_10_, VOC, and NH_3_)	NIER	Emission estimates (table)
Vegetation	Annual summary (average, minimum, and maximum) of NDVI	IIS	Satellite image (raster)
	Median value in August for previous, current and following years		
Altitude	Absolute elevation	USGS	Digital Elevation Data (raster)
	Proportion of concentric elevation points above or below 20 or 50 m		

MR1, major road 1; MR2, major road 2, TSP, total suspended particle; CO, carbon monoxide; NO_X_, nitrogen oxides; SO_X_, sulfur oxides; NH_3_, ammonia; VOC, volatile organic compounds; NDVI, Normalized Difference Vegetation Index; KTDB, Korean Transport Database; KOSIS, Korean Statistical Information Service; SGIS, Statistical Geographic Information Service; EGIS, Environmental Geographical Information Service; ODP, open data portal; SP-IDC, Shipping and Port Integrating Data Center; NSIC, National Spatial Information Clearinghouse; NIER, National Institute of Environmental Research; IIS, Institute of Industrial Science, University of Tokyo; USGS, United States Geological Survey.

a Different buffer sizes by category: traffic, 25, 50, 100, 300, 500, and 1000 m; demographic characteristics and land use, 50, 100, 300, 500, 1000, and 5000 m; emissions, 3, 15, and 30 km.

b Sum of road lengths were computed for three methods: single central lines of roads, road lines multiplied by numbers of lanes, and road lines multiplied by numbers of lanes and line widths.

**Table 2.** Clas

High spatial level	Medium spatial level
Urbanized and built area	*Residential area*^a^
	*Industrial area*
	*Commercial area*
	*Cultural, sport, recreation area*
	*Transportation area*
	*Public facility area*
*Agricultural area*	Rice paddy
	Field
	Cultivated field under structure
	Orchard
	Other cultivated field
*Forest area*	Broad-leaved forest
	Coniferous forest
	Mixed stand forest
*Grassland*	Natural grassland
	Artificial grassland
*Wetland*	Inland wetland
	Coastal wetland
*Bare ground*	Natural bare ground
	Other bare ground
*Waters*	Inland water
	Ocean water

a Six out of seven high-level classes and six medium-level classes of the urbanized and built area (bold and italic) were used in our study.

**Table 3.** Summary statistics of selected geographic variables by 25 urban background and 12 urban roadside regulatory monitoring sites in Seoul

Category	Variable^a^	Type	Urban background (n=25)				Urban roadside (n=12)			
			Min	Max	Mean	SD	Min	Max	Mean	SD
Traffic	Distance to the nearest road (m)	All roads	7	392	79	77	2	73	21	20
		MR1	219	3647	1469	913	44	3347	1466	1207
		MR2	47	709	261	210	2	226	41	61
	Sum of road length (km)^1^	All roads	0	2.7	1.4	0.6	1.2	4.3	2.3	0.9
		MR1	0	0.5	0.0	0.1	0.0	2.3	0.3	0.7
		MR2	0	1.4	0.4	0.4	0.6	2.6	1.2	0.6
	Sum of road lengthxlanexwidth (1000 m^2^)^1^	All roads	0	39.1	17.3	9.5	17.1	46	30.8	7.6
		MR1	0	5.4	0.2	1.1	0	22.8	2.6	6.8
		MR2	0	31.4	8.9	8.4	11	34	22.7	7.3
Demographic characteristics	No. of people^1^		4	13900	6624	4042	602	7717	2915	2024
	No. of employees^1^	Construction	0	971	173	233	0	1317	334	444
		Lodging and restaurant	0	2040	376	432	12	1772	815	573
Land use	The proportion of land use (%)^1^	Residential	0	93	39	25	1	85	25	22
		Forestry	0	49	5	11	0	29	3	8
Physical geography	Distance to the nearest river (m)		158	3861	1109	829	51	2805	1368	924
Emissions	PM_10_ (1,000 gg/m^3^)^2^		479	1031	688	148	515	983	704	109
Vegetation	Annual mean NDVI		141	167	148	6	140	155	144	4
Altitude	Altitude (m)		14	91	35	17	19	35	27	6
	Proportion of 5 km concentric elevation points (%)	Above 20 m	0	78	18	22	0	17	7	6
		Below 20 m	7	81	35	19	0	9	0	0

Min, minimum; Max, maximum; SD, standard deviation; MR1, major road 1; MR2, major road 2; NDVI, Normalized Difference of Vegetation Index; PM_10_, particulate matter less than or equal 10 μm in diameter.

a Geographic variables calculated within different sizes of buffers: buffer radii of 300 m (^1^) and 3 km (^2^).

## Supplementary Material

Table S1. Coordinate systems used in different data sources

Table S2. Minimum widths of roads depending on type of road, speed limit, and type of area in the Administrative Rule on the Structure and Installation of Road
