# Supplementary material for: Computation of geographic variables for air pollution prediction models in South Korea
Source: Environ Health Toxicol. 2015 Oct 23;30:e2015010. doi: 10.5620/eht.e2015010 (PMC4662093; doi:10.5620/eht.e2015010)
Supplement: Table S1. — Coordinate systems used in different data sources [file eht-30-e2015010-supple1.pdf]

Table S1. Coordinate systems used in different data sources

Data source	Geographical coordinate system	Projection	Origin		Added values to origin		Scale factor
			Longitude	Latitude	Easting	Northing	
KTDB	Bessel 1841	TM	128	38	400000	600000	0.9999
SGIS	Bessel 1841	TM	128.00289	38	200000	500000	1
IIS	WGS 1984	-	-	-	-	-	-
EGIS	ITRF 2000	TM	127	38	200000	500000	1

KTDB, Korean Transport Database; SGIS, Statistical Geographic Information Service; IIS, Institute of Industrial Science, University of Tokyo; EGIS, Environmental Geographical Information Service; WGS, World Geodetic System; ITRF, International Terrestrial Reference System; TM, Transverse Mercator
