# Supplementary material for: Computation of geographic variables for air pollution prediction models in South Korea
Source: Environ Health Toxicol. 2015 Oct 23;30:e2015010. doi: 10.5620/eht.e2015010 (PMC4662093; doi:10.5620/eht.e2015010)
Supplement: Table S2. — Minimum widths of roads depending on type of road, speed limit, and type of area in the Administrative Rule on the Structure and Installation of Road [file eht-30-e2015010-supple2.pdf]

Table S2. Minimum widths of roads depending on type of road, speed limit, and type of area in the Administrative Rule on the Structure and Installation of Road

Type of road	Speed limit (km/h)	Type of area	
		Non-urban	Urban
Highway		3.50	3.50
Non-highway	≥80	3.50	3.25
	≥70	3.25	3.00
	≥60	3.25	3.00
	<60	3.00	3.00
