# Supplementary figures and images for: Computation of geographic variables for air pollution prediction models in South Korea
Source: Environ Health Toxicol. 2015 Oct 23;30:e2015010. doi: 10.5620/eht.e2015010 (PMC4662093; doi:10.5620/eht.e2015010)

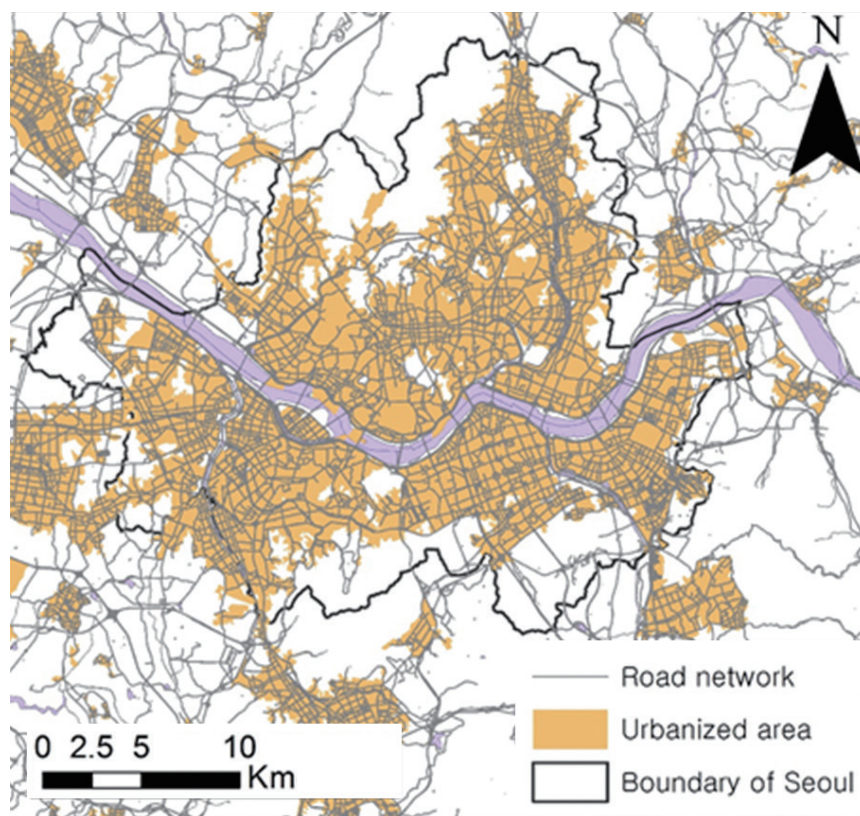

**Figure S1.** Road networks and urbanized areas in Seoul, Korea.

Supplement: Figure. S1 [file eht-30-e2015010-supple3.pdf]
